# Primary Students’ Attitudes towards Peers with Disabilities in Physical Education in Saudi Arabia

**DOI:** 10.3390/children10030580

**Published:** 2023-03-18

**Authors:** Majed M. Alhumaid

**Affiliations:** Department of Physical Education, College of Education, King Faisal University, Al-Ahsa 31982, Saudi Arabia; malhumaid@kfu.edu.sa

**Keywords:** inclusive physical education, integrated physical education, children, disability, special needs, inclusion, primary schools

## Abstract

The attitudes of students without disabilities toward their peers with disabilities are considered an important determinant of successful inclusion in physical education settings. Nonetheless, there is limited research on this topic in non-Western societies, especially in Arab contexts. Thus, to address this paucity in the literature, this study aimed to assess the general attitudes of Saudi students without disabilities towards their peers with disabilities and examine the associations between selected student-related variables (e.g., gender, age, type of school, school location, having a family member or a friend or a classmate with a disability, and having experience of playing with a person with a disability) and attitudes of students without disabilities. A total of 972 students aged 9–12 years old (M_age_ = 10.6; SD = 1.1; girls = 49.7%) completed the Arabic version of the Scale of Attitudes toward Students with Disabilities in Physical Education—Primary Education (SASDPE-PE). Data analysis indicated that, in general, participants reported positive attitudes toward their peers with disabilities in physical education classes. Despite boys being more likely to hold positive attitudes than girls, no significant difference between them existed. The results showed that 10-year-old participants reported more significantly positive attitudes than those in the other age groups. Participants attending public schools reported more significant positive attitudes toward their peers with disabilities in physical education classes compared to those attending private schools. Having a friend with a disability was linked to students without disabilities having positive attitudes towards their peers with disabilities. In contrast, having a family member or a classmate with disabilities and having played with a person with a disability were not related to such positive attitudes. The current study’s findings have significant implications for inclusive educational practices.

## 1. Introduction

Inclusion in general educational frameworks, including in general physical education settings, has been implemented globally in Sweden [1], South Korea [2], Greece [3], Japan [4], and Saudi Arabia [5]. Inclusion is the educational philosophy of teaching students with disabilities together with their peers without disabilities in the same classes and meeting the necessary and essential requirements for all students to be successful [6]. Therefore, because of this global movement to promote inclusion, the number of students with disabilities attending inclusive physical education classes has been increasing year on year [2]. Indeed, UNESCO stated that students with disabilities must be allowed to join inclusive, safe, and adapted physical education classes [7]. Although such educational policies have been implemented in general schools widely, several factors play a critical role in the success of such initiatives (e.g., stakeholders’ attitudes toward inclusion) [8,9].

Positive attitudes among stakeholders, such as students without disabilities, is an essential element to achieve successful inclusion in physical education [10,11,12]; negative attitudes remain the main obstacle in facilitating successful inclusion [13]. To explain, negative attitudes towards inclusion tend to cause students without disabilities to avoid interaction with peers with disabilities and disrupt such interactions [8]. Attitudes are defined as the emotional tendency of a person towards others, places, tasks, and objects [14]. Attitudes can be understood from a social learning perspective as the result of the interactions among individual-, environmental-, task-, and activity-related factors that all determine a person’s behavioural motivations, which then affect their outward behaviours [15]. The attitudes of students without disabilities towards students with disabilities are viewed as crucial in influencing the attitudes towards the extent to which inclusion is achievable in a particular educational context [16]. Students without disabilities have a crucial role to play in facilitating the acceptance, dignity, and respect of their peers with disabilities; having positive attitudes towards peers with disabilities appears to bolster students without disabilities’ intentions to seek to interact through play with their peers with disabilities [17]. Nonetheless, in non-Western societies, especially in Arab-speaking countries, limited attention has been paid to the roles of other significant stakeholders (e.g., students without disabilities) in terms of their attitudes toward inclusion and their attitudes towards peers with disabilities [18,19]. Understanding the attitudes of students without disabilities toward the inclusion of students with disabilities will help to address the obstacles and difficulties related to achieving successful inclusive practices [20]. Therefore, investigating the attitudes of students without disabilities toward their peers with disabilities is crucial to support successful and effective inclusion practices [21,22].

Many published studies on inclusion in physical education settings, e.g., [18,19,23,24], showed that inclusive education provides a range of benefits for students with and without disabilities. These include opportunities to learn and practice social skills, understand the importance of practicing physical exercise to promote physical fitness and motor skills, develop relationships with one another no matter the level of ability, and behave appropriately to one another [24,25,26,27]. Specifically, with respect to students with disabilities, Kodish et al. [28] found that students with disabilities (autism) did not influence the time their peers without disabilities spent on physical activity when both were educated in the same physical education classes. Additionally, as a result of such inclusion, students without disabilities learned to interact with and approach students with different abilities [29], understand student’s strengths and weaknesses [30], became more aware of the needs of students with disabilities [27], and learned more about their peers with disabilities [19]. Thus, to ensure that students with disabilities successfully attain meaningful experiences in their physical education classes, it is essential to secure the contribution of their peers without disabilities to facilitate a positive and productive context for learning [31].

There is a recently developed body of research examining students without disabilities’ attitudes towards the inclusion of peers with disabilities and the factors associated with such attitudes [9,32]. For example, a study found that students who had contact with a peer with a disability or had a family member with a disability had more positive attitudes towards students with disabilities compared to students who were simply attending an inclusive class who did not have any direct contact with peers with disabilities or had a family member with a disability [33,34]. However, the opposite was found in studies where students were attending general schools: participants without disabilities reported more positive attitudes toward their peers with disabilities than those attending inclusive schools [11,35]. This indicates that placing students with and without disabilities in the same classroom does not necessarily guarantee more positive attitudes toward peers with disabilities. Macmillan et al. [36] found in a review of the literature that out of 35 studies analysed, 22 found positive attitudes following contact with people with disabilities. The contact–attitude relationship was negative in only two studies. Moreover, Armstrong et al. [37] showed that the quantity of contacts exceeds the frequency of contacts, with positive attitudes being more related to quality contacts (e.g., having positive interpersonal experiences) with peers with disabilities rather than having frequent contact [38]. Gonçalves and Lemos [33] examined the attitudes of Portuguese students without disabilities toward students with disabilities. Their findings showed that female students had more positive attitudes than males toward their peers with disabilities. Moreover, a systematic review by Hutzler [39] reported that gender was the most significant predictor of attitudes toward inclusion where females expressed more positive attitudes toward their peers with disabilities than males. However, the impact of gender was not reported by Arampatzi et al. [35]. In Spain, Rojo-Ramos et al. [40] reported significant differences between the two research cohorts; females and students from rural schools expressed more positive attitudes toward their peers with disabilities in physical education classes. Additionally, because the type of school (i.e., public or private) may play a role in students’ attitudes towards their peers with disabilities, it is important to examine the influence of this variable. Alnahdi’s [20] study illustrated that Saudi older students without disabilities expressed more positive attitudes towards their peers with disabilities than younger students; having a relative with disabilities did not affect such attitudes. Another study, however, reported that the age of students only had a small effect on the attitudes of students without disabilities toward the inclusion of students with disabilities [41]. Additionally, the grade of students without disabilities was not significantly associated with their attitudes toward the inclusion of students with disabilities [42].

Even though there has been increasing global attention paid towards improving educational practices to benefit students with disabilities, few studies have investigated the attitudes of non-Western students (i.e., Saudi Arabian) students without disabilities towards their counterparts with disabilities in physical education contexts, as well as the factors that underpin these attitudes, leading to the following questions: (1) Do Saudi students without disabilities generally have positive attitudes toward their counterparts with disabilities? (2) What exogenous factors (e.g., gender, age, etc.) predict the attitudes of primary school students without disabilities toward their peers with disabilities in physical education? This study aimed to investigate the general attitudes of Saudi students without disabilities towards their peers with disabilities and examine the linkages between the eight target research variables (i.e., gender, age, type of school, school location, having a family member or a friend or a classmate with a disability, and having played with a person with a disability) and students’ attitudes toward their peers with disabilities in the physical education framework. Such baseline data are crucial to enable Saudi Arabian educational stakeholders (legislators, decision-makers, administrators, and teachers) to develop and modify inclusive education interventions and policies to achieve full inclusion for students with disabilities in physical education settings. Finally, it is hoped that the findings will provide novel empirical data to facilitate inclusive educational practices at the local level.

## 2. Materials and Methods

### 2.1. Participants

A total of 1000 students (boys: *n* = 500; girls: *n* = 500) were randomly selected using geographical clustering from male and female public and private primary schools in Eastern province in Saudi Arabia. Eligibility criteria included participants aged 9–12 whose parents had signed an informed consent form. After 28 responses were deleted because they were incomplete, the total sample included 972 students (M_age_ = 10.6 and SD = 1.1 years old; 488 boys M_age_ = 10.6 and SD = 1.1 years old; 484 girls M_age_ = 10.6 and SD = 1.1 years old). The independent variables were as follows: gender (male/female), age (9, 10, 11, or 12 years old), type of school (public or private), school location (urban or rural), having a family member with a disability (yes or no), having a friend with a disability (yes or no), having a classmate with a disability (yes or no), and having played with a person with a disability (yes or no).

### 2.2. Procedure

After obtaining ethical approval from the Research Ethics Committee at King Faisal University (KFU-REC-2022-AUG-ETHICS149), the participants were invited to join the study. The informed consent forms were signed by parents or legal guardians after reading the information sheet that described the aims of the study; instructions for completing the questionnaire were also provided. The questionnaire was created using Google Forms so that the participants could complete the questionnaire online by clicking the URL link. The participants were informed of their right to withdraw from the study at any time. All data were recorded anonymously.

### 2.3. Instruments

#### 2.3.1. Demographic Form

Data on the following eight factors were collected from participants: (i) gender, (ii) age, (iii) type of school (public or private), (iv) school location (urban or rural), (v) whether they had a family member with a disability, (vi) whether they had a friend with a disability, (vii) whether they had a classmate with a disability, and (viii) whether they have played with a person with a disability.

#### 2.3.2. Students’ Attitudes Scale

To examine the students without disabilities’ attitudes toward their peers with disabilities in physical education classes, the Scale of Attitudes toward Students with Disabilities in Physical Education—Primary Education (SASDPE-PE) was used [43]. The scale consists of four items: (i) I prefer not to interact with people with disabilities; (ii) I would avoid doing classwork with a person with a disability; (iii) I would prevent a person with a disability from joining my team; (iv) I would not propose a person with a disability as captain of my team. A five-point Likert scale scoring system was used for all four items; it ranged from 1 (strongly agree) to 5 (strongly disagree), and the sum of the four items was taken as the SASDPE-PE score, which reflects the participants’ overall attitudes toward their peers with disabilities. Because the four items were negatively worded, a higher score indicates more positive attitudes by students without disabilities toward their peers with disabilities. As the SASDPE-PE scale is only available in Spanish [43], and the participants in the present study only speak Arabic, it was necessary to translate it into Arabic (the official language of Saudi Arabia). The translation process was performed using the bilingual method [44] by three (Arabic and Spanish) bilingual physical education professors. The Spanish items were translated based on the meaning of each item rather than verbatim. The accuracy of the Spanish and Arabic versions was then compared, and the necessary changes were made. No item was removed during the translation process.

### 2.4. Reliability and Validity

The construct validity of the SASDPE-PE scale in Arabic was examined via principal component analysis and exploratory factor analysis; reliability was examined using Cronbach’s alpha. The SASDPE-PE scale was validated for students aged 9–12 from the original Spanish version using a sample of 87 students who have the same characteristics as the current participants. The results indicated that the Kaiser–Meyer–Olkin (KMO) sample adequacy measure was 0.782 and the Bartlett test sphericity score was statistically significant, indicating that commonalities were >0.30. The item scores were then subjected to exploratory factor analysis, which identified a single component with 72.088% of the total variance explained, confirming that the four items represented a one-dimensional construct. The load factor analysis indicated that all items in the domain made a significant contribution (>0.50: 0.753–0.918). The Cronbach’s alpha results indicated good reliability (α = 0.861) [45]; the correlational analysis found moderate and significant correlations for all items with no overlap [46]. Thus, the Arabic version of the SASDPE-PE scale was valid and reliable for assessing attitudes of Saudi students without disabilities toward students with disabilities in physical education settings.

### 2.5. Data Analysis

Indices of normality (e.g., skew, kurtosis) were investigated via the continuous variables included in the study. The variance inflation factors (VIFs) were used to test the multicollinearity problem; correlation analysis among all variables considered in the study was carried out, and dummy variables were created when necessary. To deal with the unequal sample sizes, the item score and overall attitudes score data were also tested for equality of variance using Leven’s test. A one-way MANOVA was performed for subsequent comparisons between participants stratified by gender, age, type and location of school, having a family member with a disability, having a friend with a disability, or having a classmate with a disability, and having played with a person with a disability; Bonferroni’s post hoc test was used to perform multiple comparisons [47]. A stepwise linear regression model was applied to analyse the association of the SASDPE-PE score with the significant independent variables in the first research question. Statistical analysis was performed using SPSS V.26 (IBM, Armonk, NY, USA) and *p*-values were set at 0.05.

## 3. Results

### 3.1. Attitudes toward Students with Disabilities in Physical Education

A total of 972 students completed all phases of the study, including 488 boys aged M = 10.6 (SD = 1.1 years) and 484 girls aged M = 10.6 (SD = 1.1 years). Most participants (88%) were studying at public schools and (89%) at urban schools, and 220 (22.6%), 223 (22.9%), 282 (29%), and 247 (25.4%) of them were 9, 10, 11, and 12 years old, respectively.

One-way MANOVAs were conducted to explore the group differences on the four items from SASDPE-PE and the overall score. Results showed that significant differences were found in the type of school (F = 7.129, *p* = 0.001) and in those who had a family member with a disability (F = 5.242, *p* = 0.001) or a friend with a disability (F = 3.753, *p* = 0.005). However, higher overall attitudes toward their peers with disabilities were only noted among participants attending public schools compared to participants attending private schools and among participants with who had friends with disabilities compared to those without. No significant difference was noted in the overall attitudes toward their peers with disabilities among participants who had a family member with a disability compared to those who did not. There were also no significant differences in any of the four SASDPE-PE items and in the overall score between participants stratified by gender, school location, having a classmate with a disability, or having played with someone with a disability. The MANOVA analysis also revealed significant age group differences in the overall attitudes of participants towards their counterparts with disabilities on several items (see Table 1). Specifically, compared to the other three age groups, 11-year-old participants reported less positive attitudes toward peers with disabilities in physical education classes in items 1, 3, and 4, and their overall scores; for item 2, 10-year-old participants reported more positive attitudes towards peers with disabilities compared to the other three age groups (i.e., 9-, 11-, and 12-year-olds). Regarding the items on having a family member with a disability, having a friend with a disability, having a classmate with a disability, and having played with a person with a disability, most of the participants (*n* = 772, *n* = 760, *n* = 820, and *n* = 516, respectively) responded “no”. Participants from public schools and those who had a friend with a disability were more likely to interact (F = 21.325 and 10.217, *p* = 0.001, respectively) and do class work with a peer with a disability (F = 19.109 and 10.265, *p* = 0.001, respectively). Participants who had a friend with a disability also preferred to do class work with peers with disabilities (F = 10.313, *p* = 0.001). Finally, students in public schools without disabilities were more likely to appoint someone with a disability to lead their team than those in private schools (F = 6.57, *p* = 0.011).

### 3.2. The Association of the SASDPE-PE Score with Independent Variables

Stepwise linear regression was conducted with the only significant independent variables in the first research question, namely age, type of school, and having a family member or a friend with a disability. The analysis revealed that, on average, 11-year-old students scored −1.261 points on the SASDPE-PE scale compared to their 12-year-old peers (see Table 2). However, compared to being a private school student, being a public school student improved attitudes toward peers with disabilities by 1.478 points. Finally, having a friend with a disability also improved attitudes towards peers with disabilities by 0.670 points, with an overall R-squared of 5.9%.

## 4. Discussion

This study aimed to investigate attitudes of Saudi students without disabilities towards their peers with disabilities and the association between selected student-related variables (i.e., gender, age, type of school, school location, having a family member or a friend or a classmate with a disability, and having played with a person with a disability) and attitudes of students without disabilities towards their peers with disabilities. In general, the findings indicated that Saudi students without disabilities expressed positive attitudes towards their peers with disabilities. Significant differences were evident between the participants for some variables (i.e., age, type of school, and having a family member or a friend with a disability). Stepwise linear regression also revealed that age, school type, and having a friend with a disability were significantly associated with attitudes toward peers with disabilities, with an overall R-squared of 5.9%. This low percentage is expected and does not concern us given the situation of people with disabilities in Saudi Arabia. Indeed, although the state guarantees the rights of citizens and their families in times of emergency, illness, disability and old age, there has been a growing tendency to view disability through the medical model rather than social [5]. People with disabilities are still stigmatized by their family members who associate disability with a kind of powerlessness and lifelong dependence, so the person with disability is isolated at home, excluded from social gatherings, and sometimes forbidden from family visits [48,49].

Although significant differences between the participants in the current study were not evident for some of the selected student-related variables, others are of significant interest to researchers. For example, despite no significant differences existing between male and female students, boys reported more positive attitudes toward students with disability than girls. These interesting findings are inconsistent with most previous studies, e.g., [19,33,39]. In particular, Gonçalves and Lemos [33] examined Portuguese students without disabilities’ attitudes toward peers with disabilities, and reported that female students expressed more positive attitudes than males. Nonetheless, the current study’s finding was unsurprising because Saudi girls have limited experiences of physical education class: only in 2018 did Saudi Arabia start to provide physical education classes for girls in public schools. Therefore, this variable must be taken into account when examining female students’ attitudes towards peers with disabilities in physical education contexts in Saudi Arabia.

In terms of the variable age, although its effect is not consistent across the four items, the data showed that 9- and 10-year-old students express relatively more positive attitudes toward their peers with disabilities than 11- and 12-year-old students. Although there is no agreement in the literature in this regard [8], the current study’s findings were consistent with the findings of Blackman’s [41] and more recently Di Maggio et al.’s [50] studies. The latter reported that younger Barbadian and Italian students reported more positive attitudes toward peers with disabilities than older students. However, the current study’s findings were inconsistent with those of Alnahdi [20], and more recently Li et al. [51], which reported that older students expressed more positive perspectives and attitudes toward the inclusion of their peers with disabilities than younger students. Nonetheless, several studies reported non-significant differences between students without disabilities’ attitudes toward students with disabilities based on participant age [21]. As there is no consistency on the impact of age in students without disabilities’ attitudes toward peers with disabilities, further investigation of this topic is required.

In terms of the variable type of school, the current study’s findings indicated that participants attending public schools expressed more positive attitudes toward peers with disabilities than those attending private schools. This may be accounted for by the different educational policies and curriculums of public and private schools which could play a critical role in students’ attitudes toward disability. In other words, public schools in Saudi Arabia tend to implement the policies of local education authorities and follow standard curricula set out by local government; private schools, however, are more likely to implement international educational policies and curricula which may focus on specific subjects such as the sciences. It could be also a result of the interactions between students with and without disabilities in public schools compared to private schools because the former includes more students with disabilities than the latter. Nonetheless, in line with the current study’s findings, a Saudi study by Al-Salim [52] indicated that public school students without disabilities reported positive attitudes toward their peers with disabilities in physical education settings.

The findings on the variable school location highlighted that participants attending schools in rural areas demonstrated similar attitudes toward peers with disabilities as participants attending schools in urban areas. Although there is limited research on the differences between urban and rural students’ attitudes towards peers with disabilities, a recent study by Rojo-Ramos et al. [40] found that Spanish students without disabilities attending rural schools reported more positive attitudes towards peers with disabilities in physical education settings than those attending urban schools. Moreover, in contrast to the current study’s findings, Rojo-Ramos et al. [40] reported a significant difference between the attitudes of students without disabilities in rural and urban areas towards peers with disabilities. Although Rojo-Ramos et al. [40] did not provide an explanation for this finding, in the Saudi case (as found in the current study), one potential reason that participants in rural areas report similar attitudes toward those with disabilities as those from urban areas is that the former, despite the reduced opportunity to meet or interact with many students with disabilities in their physical education classes or schools, conduct high levels of contact with their peers with disabilities [38]. Therefore, the current study’s findings were inconsistent with previous research reporting that interaction with peers with disabilities is a significant predictor of positive attitudes among students without disabilities [12]. In fact, contrary to the findings of Majoko [53], the large number of students with disabilities attending physical education classes has been identified as a significant obstacle to successful inclusion. Alnahdi et al. [54] reported that the quality, rather than the duration, of exposure was found to be the most important factor affecting attitudes towards people with disabilities. Meaningful, close, and intimate engagement with people with disabilities is essential. These authors also suggest that early intervention is necessary to improve access not only quantitatively but also qualitatively. Villages are an agglomeration of human settlements often located in rural areas with a population of between 6000 and 15,000 inhabitants [55]. Therefore, it appears logical that class sizes in rural schools tend to be very small.

Finally, the current study’s findings illustrate that participants who had a friend with a disability expressed more positive attitudes toward students with disabilities than those who did not have a friend with a disability. Friendship between students without disabilities and those with disabilities has been identified as a significant predictor of the former’s attitudes towards the latter. In line with the current study’s findings, a study by Blackman [41] reported that having a friend with a disability positively impacted students without disabilities’ attitudes toward their peers with disabilities. Campos et al. [56] indicated that students who reported having a close friend with a disability expressed their acceptance of having a classmate with a disability in their physical education classes. In support, the existence of a positive friendship between students with disabilities and peers without disabilities is more likely to benefit both groups in the physical education settings [19]. Although such friendship has a positive effect on students’ attitudes toward disability, besides friendship, several elements (e.g., type of disability and gender) may play a role in such attitudes. For instance, Olaleye et al. [57] indicated that the friendship factor did not affect boys’ attitudes toward disability, but girls who have a friend with a disability reported more positive attitudes. Thus, these results might suggest that there is a complex and multifaceted relationship between friendship and attitudes toward disability [21].

Despite its merits, two limitations should be highlighted in this study. First, the samples involved in this study were of specific ages and from a single area of Saudi Arabia; therefore, the findings may not be generalized to students of different ages and from different areas of the country. More research is needed that collects larger samples of different ages from different areas of the country. Second, the current study employed a quantitative research approach via an online questionnaire; therefore, the participants did not have the opportunity to express their in-depth feelings and attitudes towards their peers with disabilities. Further research using qualitative research methods or a mixed-methods design would help to gather more comprehensive data about students without disabilities’ attitudes toward disability.

## 5. Conclusions

The positive attitude of students without disabilities towards their peers with disabilities is one of the most significant factors in successfully supporting the implementation of inclusive physical education. In the current study, the Saudi students without disabilities expressed generally positive attitudes toward their peers with disabilities in physical education settings. These findings make us optimistic that the inclusion process in Saudi Arabia is moving in the right direction. In other words, the positive attitudes of students without disabilities towards the inclusion of students with disabilities provides a positive indicator of successful inclusion. However, these findings should be interpreted with caution because several other factors may also critically affect such attitudes (e.g., age, school type (public or private), school location (urban or rural), and having a friend with a disability). Nonetheless, the findings contribute to the literature by providing baseline data on students without disabilities’ attitudes toward their peers with disabilities, which may also help educational policy-makers in Saudi Arabia and Arab countries to enact successful educational policies and legislation more broadly that maintain pace with the rapid development of inclusion in physical education globally.

## 6. Implications

The findings of this study give rise to three main implications. First, the data show that the school is effective at increasing the likelihood that students with disabilities will be accepted by their peers without disabilities; therefore, it is crucial to expand the opportunities for such students to interact with their peers without disabilities. Those with disabilities also should be portrayed as integral members of society who simply perform certain activities differently and therefore should be included as much as possible in curricular and extracurricular activities; this will promote a better awareness and understanding of people with disabilities. Second, Saudi officials should consider expanding the inclusion of students with disabilities alongside their peers in public and private schools. All school leaders across the country are encouraged to promote ongoing inclusion-led school activities to provide all students with opportunities to meet and interact with their peers with disabilities to further the inclusion and acceptance of the latter in social activities. Third, conducting further studies that focus on different independent variables will help to better understand the relationships between individuals with and without disabilities across different age groups and promote positive attitudes towards those with disabilities from infancy through to old age.

## Figures and Tables

**Table 1:** Specific items score and the overall score of the participants’ attitudes toward disability in physical education lessons among Saudi students aged 9–12.

Tests of between-Subjects Effects							
Factors	Multivariate Tests		I Prefer Not to Interact with People with Disabilities.	I Would Avoid Doing Classwork with a Person with a Disability.	I Would Prevent a Person with a Disability from Joining My Team.	I Would Not Propose a Person with a Disability as Captain of My Team.	Score
Gender	males	N = 488	4.2 (1.2)	4.4 (1.1)	4.2 (1.1)	3.9 (1.1)	16.7 (3.7)
	females	N = 484	4.0 (1.3)	4.3 (1.2)	4.1 (1.3)	3.8 (1.4)	16.3 (4.5)
	F (4967)	1.791 (NS)	Not applicable				
	Partial η2	0.007					
	Wilk’s Λ	0.993					
Age (years)	9	N = 220	4.3 (1.1)	4.3 (1.1)	4.3 (1.1)	4.1 (1.3)	16.9 (4.1)
	10	N = 223	4.3 (1.2)	4.7 (0.7) !!♣♣♠	4.3 (1.1)	4.1 (1.2)	17.4 (2.9)
	11	N = 282	3.8 (1.4) !!¶¶♠	4.1 (1.3)	3.9 (1.4) !!¶¶♠	3.5 (1.3) !!¶¶♠	15.3 (4.7) !!¶¶♠
	12	N = 247	4.2 (1.2)	4.3 (1.1)	4.3 (1.2)	3.9 (1.3)	16.7 (3.9)
	F (12,2553.44)/F (3)	5.68 ***	8.246 ***	10.207 ***	7.752 ***	12.546 ***	12.78 ***
	Partial η2	0.023	0.025	0.031	0.023	0.037	0.038
	Wilk’s Λ	0.033					
Type of school	public	N = 856	4.2 (1.2)	4.4 (1.1)	4.2 (1.2)	3.9 (1.2)	16.7 (3.9)
	private	N = 116	3.6 (1.4)	3.9 (1.5)	4.0 (1.4)	3.6 (1.6)	15.1 (5.4)
	F (4967)/F (1)	7.129 ***	21.325 ***	19.109 ***	3.791 (NS)	6.57 *	15.901 ***
	Partial η2	0.029	0.022	0.019	0.004	0.007	0.016
	Wilk’s Λ	0.971					
School localization	urban	N = 866	4.1 (1.3)	4.3 (1.2)	4.1 (1.3)	3.8 (1.3)	16.4 (4.1)
	rural	N = 106	4.4 (1.0)	4.5 (0.9)	4.4 (1.1)	4.1 (1.1)	17.4 (3.6)
	F (4967)	1.613 (NS)	Not applicable				
	Partial η2	0.007					
	Wilk’s Λ	0.993					
Having a family member with a disability	no	N = 772	4.1 (1.2)	4.3 (1.2)	4.2 (1.2)	3.8 (1.3)	16.4 (4.2)
	yes	N = 200	4.1 (1.4)	4.6 (1.0)	4.2 (1.3)	4.0 (1.3)	16.9 (3.6)
	F (4967)/F (1)	5.242 ***	0.000 (NS)	10.313 ***	0.196 (NS)	1.436 (NS)	1.931 (NS)
	Partial η2	0.021	0.000	0.011	0.000	0.001	0.002
	Wilk’s Λ	0.979					
Having a friend with a disability	no	N = 760	4.1 (1.2)	4.3 (1.1)	4.1 (1.2)	3.8 (1.3)	16.3 (4.2)
	yes	N = 212	4.4 (1.3)	4.6 (1.0)	4.3 (1.2)	4.0 (1.1)	17.2 (3.6)
	F (4967)/F (1)	3.753 **	10.217 ***	10.265 ***	1.572 (NS)	2.681 (NS)	7.487 **
	Partial η2	0.015	0.01	0.01	0.002	0.003	0.008
	Wilk’s Λ	0.985					
Having a classmate with a disability	no	N = 820	4.1 (1.2)	4.4 (1.1)	4.2 (1.2)	3.9 (1.3)	16.6 (4.1)
	yes	N = 152	4.1 (1.2)	4.2 (1.2)	4.0 (1.3)	3.8 (1.3)	16.1 (4.2)
	F (4967)/F (1)	1.438 (NS)	Not applicable				
	Partial η2	0.006					
	Wilk’s Λ	0.994					
Having played with a person with a disability	no	N = 516	4.1 (1.2)	4.3 (1.1)	4.1 (1.2)	3.9 (1.2)	16.4 (4.0)
	yes	N = 456	4.2 (1.3)	4.4 (1.2)	4.2 (1.3)	3.9 (1.4)	16.7 (4.2)
	F (4967)/F (1)	1.926 (NS)	Not applicable				
	Partial η2	0.008					
	Wilk’s Λ	0.992					

Note: Results are presented as mean (SD); NS = not significant; * < 0.05; ** < 0.01; *** < 0.001 significant differences between the groups; !! < 0.001 differs from the 9-year-old group; ¶¶ < 0.001 differs from the 10-year-old group; ♣♣ < 0.001 differs from the 11-year-old group; ♠ < 0.01 differs from the 12-year-old group.

**Table 2:** Stepwise linear regression model for individual predictors of the attitudes toward peers with disabilities in physical education among Saudi students aged 9–12.

Model	B (Std. Error)	R^2^	*t*	95.0% Cl for B		
				Lower	Upper	VIF
(Constant)	17.457 (0.701) ***	0.059	34.419	14.253	15.977	
Age (11)	−1.261 (0.348) ***		−3.622	−1.943	−0.578	1.529
Type of school	1.478 (0.396) ***		3.729	0.700	2.256	1.013
Having a friend with a disability	0.670 (0.317) *		3.082	0.048	1.292	1.050

Note: * = <0.05, *** = <0.001.

## Data Availability

The data that support the findings of this study are available from the author upon reasonable request.
